# X-chromosome SNP analyses in 11 human Mediterranean populations show a high overall genetic homogeneity except in North-west Africans (Moroccans)

**DOI:** 10.1186/1471-2148-8-75

**Published:** 2008-02-29

**Authors:** Carmen Tomas, Juan J Sanchez, Anna Barbaro, Conxita Brandt-Casadevall, Alexis Hernandez, Mohamed Ben Dhiab, Misericordia Ramon, Niels Morling

**Affiliations:** 1 Section of Forensic Genetics, Department of Forensic Medicine, Faculty of Health Sciences, University of Copenhagen, Copenhagen, Denmark; 2 Instituto Nacional de Toxicología y Ciencias Forenses, Delegación de Canarias, Tenerife, Spain; 3 Sezione di Genetica Forense, SIMEF (Studio Indagini Mediche E Forensi), Reggio Calabria, Italy; 4 Institut Universitaire de Médecine Légale, Lausanne, Switzerland; 5 Service de Médecine Légale, Hôpital Farhat Hached, Sousse, Tunisia; 6 Institut Universitari d'Investigacions en Ciències de la Salut i Laboratori de Genètica, Departament de Biologia, Universitat de les Illes Balears, Palma de Mallorca, Spain

## Abstract

**Background:**

Due to its history, with a high number of migration events, the Mediterranean basin represents a challenging area for population genetic studies. A large number of genetic studies have been carried out in the Mediterranean area using different markers but no consensus has been reached on the genetic landscape of the Mediterranean populations. In order to further investigate the genetics of the human Mediterranean populations, we typed 894 individuals from 11 Mediterranean populations with 25 single-nucleotide polymorphisms (SNPs) located on the X-chromosome.

**Results:**

A high overall homogeneity was found among the Mediterranean populations except for the population from Morocco, which seemed to differ genetically from the rest of the populations in the Mediterranean area. A very low genetic distance was found between populations in the Middle East and most of the western part of the Mediterranean Sea.

A higher migration rate in females versus males was observed by comparing data from X-chromosome, mt-DNA and Y-chromosome SNPs both in the Mediterranean and a wider geographic area.

Multilocus association was observed among the 25 SNPs on the X-chromosome in the populations from Ibiza and Cosenza.

**Conclusion:**

Our results support both the hypothesis of (1) a reduced impact of the Neolithic Wave and more recent migration movements in NW-Africa, and (2) the importance of the Strait of Gibraltar as a geographic barrier. In contrast, the high genetic homogeneity observed in the Mediterranean area could be interpreted as the result of the Neolithic wave caused by a large demic diffusion and/or more recent migration events. A differentiated contribution of males and females to the genetic landscape of the Mediterranean area was observed with a higher migration rate in females than in males. A certain level of background linkage disequilibrium in populations in Ibiza and Cosenza could be attributed to their demographic background.

## Background

The X-chromosome has features that make it a good source of information for population genetic studies. The X-chromosome is present in a single copy in males, which makes it possible to determine the X-chromosome haplotypes in men. Compared with autosomes, the X-chromosome has lower recombination rate, lower mutation rate and smaller effective population size resulting in a faster genetic drift. In consequence, both linkage disequilibrium (LD) and population structure in the X chromosome are expected to be stronger than those in autosomes [1]. Two-thirds of the X-chromosome history has been spent in females. Thus, X chromosome polymorphisms mainly reflect the history of females. Due to recombination, X-chromosome markers in females provide a multilocus system, while the mtDNA and Y-chromosome are linked haplotypes. Thus, X-chromosome markers are valuable for population genetic studies [1].

Due to its history with constant migration movements, the Mediterranean area constitutes an challenging region for population genetic studies, both considering the whole area [2-4] and particular populations [5,6]. Only very little genetic structure has been found among populations living on the northern and eastern shores of the Mediterranean Sea by analyses of Y-chromosome STRs [3] and mtDNA [7]. The pattern may be a consequence of the Neolithic demic diffusion in this region (around 9,000 YBP) and/or a high level of gene flow in the area. The presence of a strong genetic boundary between the northern and southern Mediterranean populations, especially in the western side of the sea has been suggested [3,8-10]. Studies focused on smaller areas have also given interesting results, especially those regarding physical and/or cultural isolates such as Corsican [11] and Sardinian sub-isolates [12], Balearic populations [13-15] and Calabrian populations [16].

In order to further investigate the genetic characteristics of the populations in the Mediterranean basin, we analyzed 25 SNPs located on the X-chromosome in eleven populations from the Mediterranean area. In addition, two populations from Northern Europe and East Africa were analyzed. We carried out a wider analysis using data from the HapMap website on 21 out of the 25 SNPs used in this study (see Methods for details).

We wanted to use the X-chromosome SNPs as multilocus markers. We selected polymorphic X chromosome SNPs with a physical distance of at least 600 kb. Population history such as genetic drift and admixture has an important consequence on the degree of population LD [17]. The level of LD on the X-chromosome is expected to be higher than on autosomes. We also wanted to use these markers to study the pairwise linkage disequilibrium and multilocus association in order to obtain knowledge of the long-term and background LD in each population, because this information can be useful for studies of population genetics and the genetics of complex diseases.

We studied the informativeness of the markers selected and the sex-biased migration rate and compared the information to that of non-recombinant markers in mtDNA and the Y-chromosome.

## Results

### Intrapopulation variability

A total of 1,078 different haplotypes were found in the 13 populations analyzed by means of 25 X-chromosome SNPs. No single haplotype was shared among individuals within or between populations.

All the markers were polymorphic in all the populations studied. The minor allele frequency varied between a minimum percentage of 10.3% (X159 marker in the Moroccan population) and a maximum value of around 50.0% (widely found). The mean gene diversity (d) of the 25 markers analyzed was very homogeneous in the 13 populations sampled. They ranged from 0.437 in the Somali population to 0.461 in Iraqis and Tunisians. Taking into account that the maximum level of expected heterozygosity for biallelic markers is 0.5, a high degree of gene diversity was found for the 25 SNPs selected in all the populations analyzed.

Among the 300 pairwise comparisons between the 25 SNP markers in the populations, between 10 (Somalia) and 23 pairwise comparisons (Ibiza) showed significant p-values (p < 0.05). However, after Holm-Sidak correction, only two pairwise LD values were significant (X085-X159 in Cosenza, p < 0.001, and X018-X029 in Sicily, p = 0.02). Nevertheless, when multilocus tests of associations were performed using all the 25 markers, the null hypotheses of linkage equilibrium were rejected in two populations: Ibiza (p < 0.01) and Cosenza (p < 0.05). Both populations present some peculiarities that may explain the existence of a higher degree of background linkage disequilibrium (see Discussion).

### Interpopulation differentiation

#### AMOVA and pairwise F_st _values

Table 1 shows the results of AMOVA analyses in 11 Mediterranean populations based on 25 X-chromosome SNPs. A total of 99.61% of the global variation was due to the diversity within populations while only 0.39% of the variation was due to interpopulation variability. The F_st _was 0.004 (p = 0.001). When the Moroccans were excluded, the significance level increased to p = 0.05. We also performed a similar statistical analysis in the nine "worldwide" populations (see Methods) by using 21 out of the 25 X-chromosome SNPs (Table 2). A ten times higher F_st _value (0.045) was observed (p < 0.001). No significant difference was found among the three European populations or between the Chinese and the Japanese population, while a high differentiation was observed among the three African populations, especially between Moroccans and Sub-Saharans.

**Table 1:** AMOVA and fixation index, F_st_, calculated in 11 "Mediterranean" populations. Calculation based on 25 X-chromosome SNPs

Source of variation	**d.f.**^a^	Sum of squares	Variance components	Percentage of variation
**Among populations**	10	74.370	0.022 Va^b^	0.39
**Within populations**	883	4995.845	5.658 Vb^c^	99.61

**Total**	893	5070.215	5.680	

F_st_: 0.004

p-value: 0.001

^a ^Degrees of freedom.

^b ^Sum of squares deviations among populations. ^c ^Sum of squares deviations within populations.

**Table 2:** AMOVA and fixation index, F_st_, calculated on 9 "worldwide" populations. Calculation based on 21 X-chromosome SNPs

Source of variation	**d.f.**^a^	Sum of squares	Variance components	Percentage of variation
**Among populations**	8	141.884	0.224 Va^b^	4.49
**Within populations**	531	2533.301	4.771 Vb^c^	95.51

**Total**	539	2675.185	4.995	

F_st _: 0.045

p-value < 0.001

^a ^Degrees of freedom.

^b ^Sum of squares deviations among populations. ^c ^Sum of squares deviations within populations.

When a locus by locus AMOVA was performed in the Mediterranean group, only three out of 25 markers showed significant (p < 0.05) variations among the Mediterranean populations (X029, X062, and X121), while 17 out of 21 markers were important for the differentiation of the 9 "worldwide" populations: X018, X029, X036, X046, X047, X056, X059, X062, X076, X085, X109, X121, X122, X131, X135, X143 and X165. The markers X004, X142, X159 and X175 were not included in these analyses due to the lack of information in the populations obtained from the HapMap Project. No correlation (data not shown) was found between the mean gene diversity and the level of differentiation among populations, as has been suggested by others [18].

**Figure 1:**
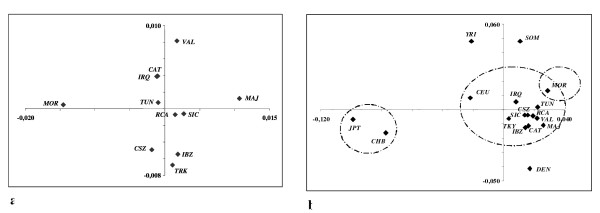
**Principal coordinate analyses plotted from pairwise Reynold's F_st _values**. a) The Mediterranean group analyzed by using 25 X-chromosome SNPs. b) All the 17 populations were represented, including those from the HapMap collection. Twenty-three X-chromosome SNPs were used. CEU: Utah residents with ancestry from western and northern Europe, DEN: Denmark, CHB: China, JPT: Japan, SOM: Somalia, YRI: Nigeria, CAT: Catanzaro, CSZ: Cosenza, IBZ: Ibiza, IRQ: Iraq, MAJ: Majorca, MOR: Morocco, RCA: Reggio di Calabria, SIC: Sicily, TUN: Tunisia, TKY: Turkey, VAL: Valencia

In order to obtain a graphical representation of the genetic structure of the populations studied, a principal coordinate analysis (PCoA) was made from the pairwise Reynold's F_st _matrix obtained using 25 or 21 X-chromosome SNPs in the Mediterranean (Figure 1a) and the 17 populations (Figure 1b), respectively.

In Figure 1a, the first two axes accounted for 39% of the total variability (first axis: 25%, second axis: 14%). Thus, the Mediterranean populations are quite homogeneous. Equivalently to the results of the AMOVA analysis, the Moroccan population was considerably displaced in the first axis, and the Majorcans were placed in the opposite direction. In the second axis, the differentiation was less, but it is interesting that the Cosenza and Ibiza populations were located opposite to their neighbouring populations (i.e. Catanzaro and Valencia, respectively). Figure 1b shows the relative position of the Mediterranean populations in relation to a wider geographical area. The first two axes accounted for 51% of the global variability (first axis: 38%, second axis: 13%). The first axis displayed East Asian populations (Japanese and Chinese populations, which did not show a significant pairwise F_st _value) opposite to the others (Middle East, European and African populations). In the second axis, the Mediterranean populations plus the CEU population (Utah residents with ancestry from northern and western Europe) formed a group between the North-European and African populations. Only the Moroccans showed a slight displacement from the others showing significant F_st _values in most of the pairwise comparisons inside the Mediterranean group. The CEU population showed an intermediate position between the North of Europe, represented by the Danish population, and the Mediterranean group. None of the pairwise comparisons between the CEU population and the Mediterranean or Danish populations showed a significant F_st _value. For the grouping proposed in Figure 1b, 4.58% of the variation could be explained by differences between groups (p < 0.001). A more detailed hierarchical AMOVA analysis showed that 8.86% of the global variation could be explained by the East Asian populations (Japan and China; p < 0.001), 3.49% of the variation was explained by Somalis and Sub-Saharans (in this case the significance level raised up to p < 0.05 due to the high heterogeneity between these two populations). The Mediterranean group (which included the CEU population) contributed to 1.33% of the global variation, a value that went down to 0.72% when the Moroccan population was excluded from the group (p < 0.001 in both cases). Finally, the North-West European populations (represented by CEU and Danish populations) only explained 0.77% of the global variation (no significant p-value).

#### Structure analysis

Additionally to the AMOVA analysis, a cluster analysis was performed using the STRUCTURE [19] software in order to assign individuals to a number of K populations allowing admixture. We estimated the posterior probability (P(X|K), where X represents the genotypes) for K between 1 and 6 for 17 populations (13 analyzed in this study and 4 from the HapMap project) using 21 X-chromosome SNPs. We observed that the proportions of the individuals assigned to each K subpopulation were balanced in all cases indicating that the X-chromosome SNPs did not identify to which subpopulation an individual belongs. According to the authors of the programme [19], this could be interpreted as a lack of population structure as a consequence of a high level of admixture.

Due to the statistical model implemented in STRUCTURE, it is possible to find significant F_st _values without finding significant differences using STRUCTURE when the populations analyzed are closely related [19]. This is not the case of some of the populations included in the analysis. As it was discussed by other authors [20], it could be that the high migration rate of females could have resulted in a small genetic structure in X-linked loci. On the other hand, a lack of resolution of the investigated SNP markers could also be the cause of the low level of genetic structure observed.

#### Isolation by distance

**Figure 2:**
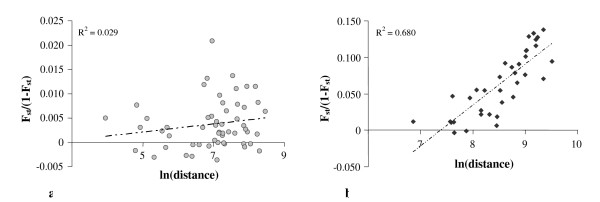
**Pairwise differentiation versus the natural logarithm of the distances (in km) following Rousset's two-dimensional isolation by distance model**. a) Plot based on the analyses of 25 X-chromosome SNPs in 11 Mediterranean populations: *F*_*st*_/(1 - *F*_*st*_) = 0.001*ln(*dist*.) - 0.004. b) Analysis carried out on 9 worldwide populations and 21 SNPs: *F*_*st*_/(1 - *F*_*st*_) = 0.062*ln(*dist*.) - 0.470.

We did not find any significant correlation between genetic differentiation (calculated as F_st_/(1-F_st_)) and the natural logarithm of the geographical distance (in kilometres) in the Mediterranean area based on the study of 25 X-chromosome SNPs (Figure 2a). According to Rousset's isolation-by-distance model [21], some demographic information can be obtained from the model drawn (Figure 2a). An indirect estimation of 4D μσ^2^, also known as the "Neighbourhood size" (where D is population density and σ^2 ^the dispersal variance), could be calculated from the slope value (0.001). Considering the 25 biallelic markers analyzed in the present work, the estimated value of 4D μσ^2 ^would be around 935 individuals, which seems to be an underestimated value according to the same author. This data can be interpreted as the result of a high number of migration events in the recent history of the human populations in the Mediterranean basin.

Figure 2a reflects a strong impact of gene flow versus genetic drift in the Mediterranean region (according to Koizumi et al [22]). Nevertheless, it is worth to pay attention to specific populations where other demographic scenarios such as geographic isolation, genetic drift or population admixture have probably played an important role in their current genetic constitution. As we have commented above, relative high genetic distances between neighbouring populations were found between Cosenza and (1) Catanzaro and (2) Reggio di Calabria and between Ibiza and (1) Majorca and (2) Valencia. The results support the hypothesis that the genetic differentiation of the Ibiza and Cosenza populations is a result of their particular demographic histories [15,16,23]. The Moroccan population showed a high level of differentiation in relation to the other populations, even to those that are geographically close to Morocco (Western side of the Mediterranean basin).

The number of estimated migrants was dramatically reduced when a much wider area was considered (Figure 2b, where 9 populations were taken into account: 3 African populations (from Morocco, Nigeria and Somalia), 3 Asian populations (from Iraq, China and Japan) and 3 European populations (from Denmark, CEU – Utah residents with ancestry from northern and western Europe- and South Italy). The analyses of populations belonging to an extended area are more prone to reflect more ancient demographic processes [24]. If the Neolithic transition occurred around 9,000 YBP, and human dispersal was close to 30 km per generation, pre-agricultural migration patterns could be observed at distances over 1,000 km [24]. Taking this information into account and the fact that the minimum geographic distance between two populations in Figure 2b was 954 km, pre-agricultural migration events should be reflected there. On the basis of 25 X-chromosome SNPs, an indirect estimate of 16 migrants between subpopulations was calculated from the regression slope (R^2 ^= 0.680, p < 0.001 Spearman rank correlation coefficient). In this study, isolation by distance operated better at large geographic scales than at short distances.

### X-chromosome SNPs vs. non-recombinant systems (mtDNA HVRI and 35 Y-chromosome SNPs)

#### Mantel test

We performed two Mantel tests for each of the three sets of markers: 25 X-chromosome SNPs, HVRI mtDNA and 35 Y-chromosome SNPs (Table 3). First, we analyzed 11 of the 13 populations in this work for which results of all the markers were available. Then, only the Mediterranean populations were analyzed (nine populations). Mantel correlations were corrected for geographical distances by computing partial correlations between genetic distances at constant geographic distance (see Methods). All tests were significant (p < 0.05) except the test between X-chromosome and mtDNA in the Mediterranean area, where the correlation index was low (rY1_2 = 0.190) but still positive. The lack of significance was most likely due to the low degree of genetic differentiation of both X-chromosomes and mtDNA in the area. All X-chromosome and mtDNA markers showed a significant correlation (p < 0.05) with Y-chromosome SNPs both when 9 or 11 populations were analyzed. Except in particular cases (e.g. Ibiza [25]), male and female movements in the Mediterranean have apparently had the same overall tendency. An important difference in the intensity of the migration events of both sexes could also be seen in the level of significance of the different Mantel tests.

**Table 3:** Partial Mantel correlation between pairwise distances matrices obtained from three sets of genetic markers at constant geographical distance (r((set of markers 1, set of markers 2), geographical distance)).

	X-SNPs vs mtDNA	X-SNPs vs Y-SNPs	mtDNA vs Y-SNPs
**11 populations**^a^	*rY1_2 *= 0.653	*rY1_2 *= 0.557	*rY1_2 *= 0.577
	*p *= 0.024	*p *= 0.004	*p *< 0.013
**9 populations**^b^	*rY1_2 *= 0.190	*rY1_2 *= 0.556	*rY1_2 *= 0.587
	*p *= 0.233	*p *= 0.020	*p *= 0.004

^a ^Populations: DEN, IBZ, IRQ, MAJ, MOR, RCA, SIC, SOM, TUN, TRK and VAL.

^b^Populations: IBZ, IRQ, MAJ, MOR, RCA, SIC, TUN, TRK and VAL.

For abbreviators see Figure 1.

#### Isolation-by-distance analyses by using different genetic systems

**Figure 3:**
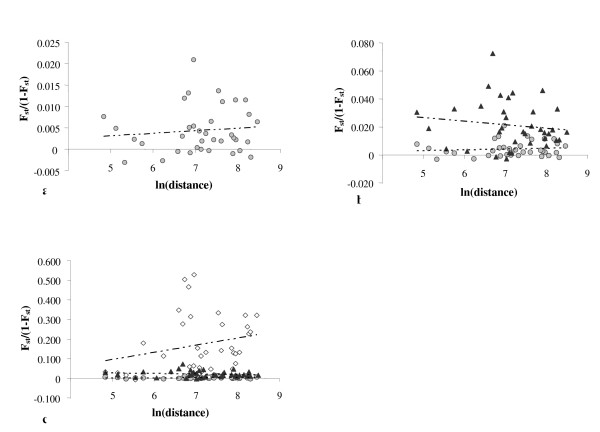
**Two-dimensional isolation-by-distance plot**. Representation of 9 populations from the Mediterranean region analyzed by three different markers: a) 25 X-chromosome SNPs (grey circles); b) HVRI mtDNA (black triangles) compared to X-chromosome; c) 35 Y-chromosome SNPs (empty rhombuses) compared to mtDNA and X-chromosome.

Figure 3 shows a plot of a two-dimensional isolation-by-distance model based on three sets of genetic markers in populations in the Mediterranean area (due to the lack of mtDNA data of the population from Catanzaro and Cosenza, these populations were not included in the analyses).

As expected from previous studies [3,26], no significant correlation between genetic and geographic distances was found in any case. From a demographic point of view, the estimated number of migrants based on X-chromosome results would be approximately the same as calculated above (971 individuals), and around 32 were calculated from Y-chromosome SNP data. Surprisingly, a negative slope was obtained in the case of the mtDNA, reflecting a major effect of isolation and genetic drift versus migration in certain populations of the Mediterranean area.

When X-chromosome SNPs and mtDNA were analyzed in a wider geographic area (data not shown), a greater female migration rate compared to the male migration rate was still observed. As it has been commented above, the minimum geographic distance between two populations was approximately 954 km and mainly pre-agricultural migration events were then represented in this case.

## Discussion

A considerable amount of information based on classical genetic markers [8,27,28], autosomal STRs [26], mtDNA [2,7] and Y-chromosome haplotypes [3,4,10] has previously been reported concerning the genetics of populations in the Mediterranean region. A low level of genetic structure has been described due to the high degree of migration in the Mediterranean area [3]. Apparently, the surrounding lands of the Mediterranean Sea were settled by people from the Middle East approximately 40,000 years ago [5]. The migration events increased during the Neolithic era (around 9,000 YBP) with the transition to agriculture. The increase in migration events during the history constitutes the main cause of the genetic homogeneity in the area. Several authors [9,10] have suggested the existence of a north-south genetic "barrier" in the western part, and no consensus on the current genetic landscape has been established [4,29,30]. Some isolated populations with deviating genetic differentiation have been described [5,6,16,23].

Based on the study of 25 X-chromosome SNPs, we found a low value of population differentiation (F_st _= 0.004) in 11 Mediterranean populations. The fixation index was significant (p = 0.001) but the p-value increased to p = 0.5 when the Moroccan population was excluded from the analysis. In contrast, a 10 times higher F_st _value (p < 0.001) was observed when 9 populations from three continents (3 from Europe, 3 from Africa and 3 from Asia) were analyzed. Not surprisingly, the strongest genetic differentiation was found among the 3 African populations. It is well known that African populations have a higher level of genetic heterogeneity compared to European or Asian populations [31].

A certain bias of the results obtained could be due to the fact that the markers initially were selected for forensic purposes with a high level of polymorphism in different ethnic groups. A high level of intrapopulation variability will to some degree be associated with a low interpopulation variability [3]. Nevertheless, the significant F_st _value found in the Mediterranean populations and the highly significant fixation index observed when distant populations were compared documents the suitability of these markers for population genetic studies.

### Geographic "barrier", genetic isolate and population admixture

The analysis of the Mediterranean populations in comparison to the "worldwide" populations showed a clear intermediate position between African and North-European populations with Morocco somewhat closer to the African populations than the other Mediterranean populations. When only the Mediterranean populations were analyzed in a principal coordinate plot, opposite positions of Moroccans and Majorcans were observed (Figure 1). This supports the existence of a north-south gene flow "barrier" in the western part of the Mediterranean area [9,10]. It could be argued that the genetic differentiation of the Moroccan population was a result of the arab-berber sub-structure of the individuals sampled in this work, but this hypothesis does not seem likely. Several authors [26,28] reported a high genetic homogeneity between berbers and arabs in NW Africa, so they suggested that the Arabisation of this area was probably a cultural phenomenon, which did not imply a replacement of the ancestry population. Our results give support the hypothesis of an early settlement of NW Africa [26]. The original berber population seem to have received a low genetic influx from the surrounding areas. Different hypothesis have been suggested to explain the genetic differentiation of the Moroccan population. An initial genetic drift [26,30] could have caused differences in allele frequency distribution that have not been re-established due to a certain level of geographic isolation. The Strait of Gibraltar has been described by several authors [9,10] as an important genetic barrier. Even a certain level of genetic exchange probably occurred between NW Africa and the South of the Iberian Peninsula [10,27,32,33], sharp frequency changes have been described in this area [10,33]. Also the Sahara desert has been suggested as responsible of the genetic isolation of NW African populations from Sub-Saharan populations [30]. There is no consensus about the impact of the Neolithic demic diffusion in the Mediterranean area [29,30,34]. According to our results, a low impact of the Neolithic expansions and/or later migration events on NW African populations would have occurred. Nevertheless, the high genetic homogeneity observed and especially the low level of genetic pairwise differentiations between the Iraqi and the Western Mediterranean populations (such as the East of Spain and South of Italy) supports the impact of several migration events [35] during the history in the genetic constitution of the Mediterranean area, where sailing at the Mediterranean Sea have been an important connection between various geographical areas and populations [33]. The high genetic homogeneity of X-chromosome markers contrasts with the genetic structure of Y-chromosome SNPs [33]. This highlights the differences between male and female migration rates, and stresses the importance of combining results obtained from different types of genetic markers.

Tunisians did not show a significant level of differentiation with northern populations as mentioned by others [3]. A discordant result may be due to the different nature of the markers used in the different works (e.g. mutation rate, recombination, sex specific inheritance). Also the heterogeneity described in Tunisian Berbers [36] could explain that studies carried out using different sets of Tunisian samples may end up showing different results.

More recent demographic events that took place in some particular populations in the area showed interesting exceptions to the general homogeneity. This was the case of the Cosenza and Ibiza populations that were placed relatively "far" from their neighbouring populations in the various statistical analyses performed.

Tagarelli et al [16] described the Calabria province as a collection of many "human genetic isolates". In contrast to Catanzaro and Reggio di Calabria, Cosenza did not suffer destructive telluric events that would have modified its population structure. So, the population heterogeneity inside Cosenza is more patent than in any of the other two populations. This is reflected by the heterogenic distribution of various genetic markers at the coast compared to the internal areas [16]. The results obtained with the 25 X-chromosome markers were in agreement with the previous studies. Cosenza showed not just a relatively high genetic differentiation compared to the populations from Catanzaro and Reggio di Calabria but also a certain displacement in relation to other Mediterranean populations. This was also the case of the Ibiza population. Ibiza, one of the three major islands of the Balearic archipelago, was reproductively isolated for centuries, their population effective size was reduced by infectious diseases and a high number of consanguineous marriages were reported in the 15^th^-17^th ^centuries [25]. In 1970s, the tourist influx considerably increased the population of Ibiza [23]. Former studies have emphasized the genetic differentiation of this population as a consequence of the stochastic events that happened during its history [15,23,25]. Moreover, when a parametric multilocus association analysis was performed, significant values of associations were only observed in the Ibiza and Cosenza populations. In most human populations, high levels of linkage disequilibrium do not exist between markers separated >3 kb [37]. Taking the distance (0.6–145 Mb) between the markers selected into consideration, we did not expect to find significantly increased levels of LD in any of the populations studied. On the other hand, it is well known that different demographic scenarios could result in unexpected levels of LD over large genomic regions [38]. 

#### Sex-biased migration rate: a comparative study among different parts of the genome.

Sex-biased migration rates vary between populations as well as through population history. Matrilocality results in a mtDNA geographic structure while patrilocality results in a genetic structure in the non-recombinant region of the Y-chromosome [24]. The X-chromosome, which has spent 2/3 of its history in females, reflects matrilocality to a higher degree than patrilocality.

In the light of our findings it can be said that the migration rate was higher in women than in man, both when our results were analyzed with the Mantel test and under Rousset's isolation-by-distance model. The regression slope obtained from mtDNA (with a negative value) and X-chromosome markers were smaller than those obtained from Y-chromosome SNPs. The negative slope obtained from mtDNA data can be explained by the fact that populations geographically close to each other showed a relatively high genetic distance. In accordance with the findings of other authors [39], the female migration rate was greater than that for males (in this case, in the Mediterranean area). When a much wider area was included (which usually reflects ancient demographic events), the female migration rate was still larger than the male migration rate (data not shown). Thus, we did not find any indication of a change from matrilocality to patrilocality from pre- to post-agricultural societies as it has been suggested by others [24].

## Conclusion

In summary, a general genetic homogeneity of the X-chromosome SNPs was observed in a number of populations in the Mediterranean area. The genetic distance between populations in the Middle East and the western part of the Mediterranean area was very low, most likely reflecting the effect of the Neolithic Wave and recent migration events. Only the Moroccan population showed a significant genetic distance from the remaining Mediterranean populations including populations that are geographically close to it, showing the importance of the Strait of Gibraltar as a geographic barrier and supporting the idea of a low impact of the Neolithic demic diffusion and more recent migrations in North-West Africa. In Ibiza and Cosenza populations, interesting differentiations from their neighbouring populations and significant multilocus associations were observed when all the 25 X-chromosome SNPs were analyzed reflecting the particular demographic histories of these populations. A deeper study of LD in these two populations could reveal interesting results for disease association studies.

A higher migration rate was observed in females than in males in both the Mediterranean area and in a wider geographic area showing a greater genetic flow mediated by females than males probably both in pre-agricultural and post-agricultural societies. As it was suggested by other authors [24], patrilocality seems to have been common in post-agricultural populations. According to our results, patrilocality should have also been important in pre-agricultural societies. The negative slope obtained in Rousset's isolation-by-distance model using mtDNA data in the Mediterranean area emphasizes the effect of genetic drift and genetic isolation observed in females in some of the populations in the western part of the Mediterranean Sea.

## Methods

### Population sampled

**Figure 4:**
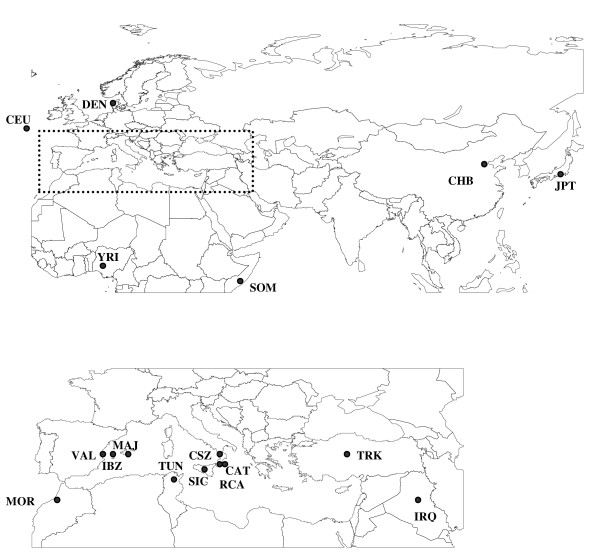
**Geographical location of the populations included in this study**. For abbreviators see Figure 1.

We analyzed a total of 1,078 unrelated males from 13 populations (Figure 4). Eleven of these live around the Mediterranean basin: Catanzaro (61 males), Cosenza (37), Reggio di Calabria, (100) and Sicily (119) from the South of Italy; Valencia (60), Ibiza (108) and Majorca (100) from the East of Spain; Tunisia (100); Morocco (89); Turkey (57) and Iraq (63). The last two samples studied as out-groups were from Denmark (93) and Somalia (91). Nearly all DNA samples were purified from blood samples using QIAamp DNA blood mini kit (Qiagen). DNA from Tunisians was investigated with blood collected on FTA cards. The protocols were approved by the Danish local ethical committee (KF-01-037/03).

For further comparisons, data from males from the International HapMap Project were downloaded (Figure 4): 30 males from "Europe" (CEU: Utah residents with ancestry from Northern and Western Europe), 22 from China (CHB: Han Chinese in Beijing), 22 from Japan (JPT: Japanese in Tokyo) and 30 from Sub-Sahara Africa (YRI: Yoruba in Ibadan, Nigeria). We collected only data from males to make the comparison to our data easy. Depending on the population, data from between 21 and 23 out of the 25 X-chromosome SNPs analyzed in this work were obtained (data from markers X004 and X175 were not found for the CEU group, and results on X142 and X159 were not accessible for any of the four populations).

### X-chromosome SNP selection and typing

Twenty-five X-chromosome SNP markers spaced across the chromosome were selected from the NCBI SNP database. The selected markers provide 300 pairwise comparisons over distances from 0.6 to 145 Mbp. Table 4 shows the location of the SNPs chosen. The criteria followed for marker selection were the following: i) High level of polymorphism in different ethnic groups, and ii) not in or close to a coding region, in order to minimize selection phenomena.

**Table 4:** X-chromosome markers

				**LOCATION**^**b**^		**DISTANCE**^**c**^
				
CODE	**rs NUMBER**^**a**^	ALLELES	REGION	bp	cM	Mbp
X004	rs2056688	A/G Forward	Xp22.3	3,463,191	007.8–007.9	1.6
X009	rs2128519	C/T Forward	Xp22.3	5,013,412	010.4–010.5	6.2
X018	rs1534285	A/G Forward	Xp22.3	11,178,926	019.2–019.3	6.2
X029	rs763056	A/G Forward	Xp22.2	17,353,443	027.5–027.6	7.6
X036	rs1373592	A/G Forward	Xp22.1	24,981,639	041.7–041.8	8.3
X046	rs993010	A/G Forward	Xp21	33,293,733	049.6–049.7	1.5
X047	rs1557054	A/G Forward	Xp21	34,834,831	051.4–051.5	4.9
X056	rs1243792	C/T Forward	Xp11.4	39,716,166	064.4–064.5	2.0
X059	rs925178	A/G Forward	Xp11.4	41,692,761	068.2–068.8	4.8
X062	rs1207480	C/T Forward	Xp11.3	46,512,334	080.0–080.1	18.9
X076	rs1936313	C/T Forward	Xq12	65,433,873	092.8–092.9	11.0
X085	rs1977719	C/T Forward	Xq13	76,438,402	096.1–096.5	16.1
X108	rs1372687	A/C Forward	Xq21	92,501,309	106.9–107.0	0.6
X109	rs1857602	C/T Forward	Xq21	93,150,340	107.8–107.9	9.2
X121	rs985425	C/T Forward	Xq22	102,300,968	117.2–117.3	2.3
X122	rs933315	A/G Forward	Xq22	104,631,674	118.3–118.4	9.9
X131	rs2190288	G/T Forward	Xq24	114,496,852	126.8–126.9	3.3
X134	rs1991961	G/T Forward	Xq25	117,802,044	139.3–139.4	1.0
X135	rs1931662	G/T Forward	Xq25	118,816,972	140.4–140.5	5.5
X142	rs149910	G/T Forward	Xq25	124,270,150	147.7–147.8	1.9
X143	rs1573704	G/T Forward	Xq26	126,204,493	149.3–149.4	11.7
X159	rs1340718	A/G Forward	Xq27	137,864,963	162.3–162.4	4.1
X165	rs1930674	C/G Forward	Xq27	141,948,792	176.9–177.0	1.0
X168	rs1339597	A/G Forward	Xq27	142,926,858	178.7–178.8	6.0
X175	rs1981452	G/T Forward	Xq28	148,898,241	193.3–193.4	-

^a ^GenBank rs number. ^b ^Approximate locations of the markers from Xp according to *Généthon *linkage map.

^c ^Approximate physical distances between two consecutive markers.

A 25-plex PCR reaction was developed in order to amplify all the DNA-fragments with the SNP markers in a single reaction, and a 25-plex minisequencing reaction was also developed. Table 5 shows the sequences of the PCR amplification primers, and Table 6 shows the sequences of the minisequencing primers. The primers were designed from GenBank sequences in the NCBI. The laboratory procedures have been previously described in detail [40]. Briefly, a 25-plex PCR amplification was followed by a multiplex SNaPshot reaction that extends a single base using fluorochrome-labelled ddNTPs. The resulting fluorescent fragments were analyzed by capillary electrophoresis using an ABI 3100 Genetic Analyzer (Applied Biosystems). PCR primers were designed to give amplicon lengths in the range of 62 to 120 bp.

**Table 5:** PCR primers for the 25 X-chromosome SNPs

CODE	FORWARD PRIMER	REVERSE PRIMER	AMPLICON SIZE (bp)
X018	CACTGGTTATTTTCTTCTTCCCTTC	CCACAAGACTAAGCCAAGACCA	62
X076	CAAACTCTGGAGACACAGATCATAC	CCCCATGAGAACTGCAATATGT	66
X036	ACACATGGGTTTTGAGTCAGC	CCATAAGGCCAATGAGTTGTAGAG	67
X004	GGGAACTGTAGCTTGATTTGTTTTC	CACCTAGAAAGCAGTCTGTGGAAC	70
X009	GAGCAGGAGAAAATCATCTGAGTA	AAGCTCTGTGTTACATTCTGTTTCC	70
X121	CTGAAACTTTCGAATCTTCTCTCAC	ATCAGGGAAGACATCTGATAAGACC	71
X159	TCATCTGAAACATAGACTAAAGTGGAA	GAAAGAAACATCTTCCTTCTGTTATTTG	72
X108	ATAAAGAGCCCATAAATCCCTGA	TCTGATTTCTGTAATTTTTCGTGGT	81
X029	CGTGCATTGGTTCTTCAGTC	CTTTTCGTGGTCCCCTCA	85
X134	AGAGGCTTGAAGAATCATAGGTTG	GAGCTAGGATCATCTGACTAAGTGG	89
X047	AACATGTCAGTGTACTTTCATAAGTTGTTA	AGATCATGTTGACATTAGGTTGCT	90
X175	ATTCAGAGATGAGATGGGAGTAGAG	CCCTCCACATCTTCAAAACCT	91
X062	CAATAGTTCAGTTGTTTCTCAGTAGAGC	TCATGCTTCTTGATTCTAGTGACTG	93
X085	AAATGAGTCAAAATGTCTCCTTCA	ATATATGCAGCTTATTTGGTGGAAG	94
X165	ATAAGTTCAGTACAGTGAATTGACAGAATA	TTCTTCAAAGTGAAATTCTTTGCTC	97
X056	GAGATAAAACTCCAGGCAGAGC	CATCCTCACAAAGAAGAGATCGT	100
X142	AAGGCCAGTTAAGTCAGTATTGTGT	GCAAGCTCATATATAGATCCATTGTTCA	100
X135	TCAGCATTTCTATAGCCCTTATCAC	TCCAAACTATTCCTCTTAGCCTTCT	106
X059	TATCCTTGCTCCACTCCTTTATCAG	GCAACTAACACCTTTGAAAGATAAGA	108
X131	TTGTTGGATGTTATCTGTCATTGAT	CTTCACTGGAAATGCAAATTGATA	109
X109	GAACATGGCAAATTTCTTTTCCTC	ATAGCATGCCTATGAATAGACCAA	117
X168	AGCAAAGTCAGAAAGATAAATGTTACAC	ATTCTTCCTTGTTGAGAATTTTCCT	118
X046	CTGAGGTATAACATGGGCAATGA	ACACTCCCTGGTTTTTAGGAAAG	119
X122	TTACCCTTAAGGTCAGGAGTAGAGTC	GGATAGTGTCCTCTGGTAGGTATTG	119
X143	AGTGGTTTTCTATTTTTCTGGTTGG	GCTTATTTTATATCTTATTCCCTCAGAGAC	120

**Table 6:** Minisequencing primers used to analyze the 25 X-chromosome SNPs

CODE	SIZE (bp)	SNP	ORIENTATION	PRIMERS
X047	18	C/T	Reverse	GTTGACATTAGGTTGCT
X108	25	G/T	Reverse	aGTGGTTATGTAAGATTTTAGCAT
X142	25	A/C	Reverse	aATCCATTGTTCATTTCAAGGTAT
X135	29	G/T	Forward	ATTTTAAAATATGCAAAGACCTTTATCT
X036	33	C/T	Reverse	agtctgacaaAGTTGTAGAGCTCAGATTGTAA
X159	33	A/G	Forward	tgacaaACTAAAGTGGAAATAAATAGCTTTTG
X131	37	G/T	Forward	agtctgacaaTTAGTATTAAACATGATTTAGCGCAT
X175	37	A/C	Reverse	tcgtgaaagtctgacaaCTCCACATCTTCAAAACCT
X018	41	C/T	Reverse	cacgtcgtgaaagtctgacaaCAAGACCAAACATTGCAAG
X062	41	A/G	Reverse	gtcgtgaaagtctgacaaATGCTGATGACTTGATTTCATT
X121	45	A/G	Reverse	ccacgtcgtgaaagtctgacaaAAGACATCTGATAAGACCTTTG
X046	45	C/T	Reverse	cacgtcgtgaaagtctgacaaTTTACCTGTGCTCGTAAATTTCT
X076	49	C/T	Forward	ctaggtgccacgtcgtgaaagtctgacaaACAGATCATACAAGCACCA
X122	49	A/G	Forward	ctaggtgccacgtcgtgaaagtctgacaaGGAAGACCAAAAACAAACA
X004	53	C/T	Reverse	aactaggtgccacgtcgtgaaagtctgacaaTAGAAAGCAGTCTGTGGAACA
X168	53	A/G	Forward	ctaaactaggtgccacgtcgtgaaagtctgacaaGAAATCCGACAAAGCAAT
X165	57	C/G	Reverse	ctaggtgccacgtcgtgaaagtctgacaaCTTAACTGATAACCAAGTCATTTGTAT
X056	61	A/G	Reverse	tcaactgactaaactaggtgccacgtcgtgaaagtctgacaaCCAGGGACCCAAACTCTT
X059	65	C/T	Reverse	ctcaactgactaaactaggtgccacgtcgtgaaagtctgacaaGAAAGCCTTCATGCTGCAATG
X109	69	A/G	Reverse	tctctctcaactgactaaactaggtgccacgtcgtgaaagtctgacaaCATGCCTATGAATAGACCAA
X134	73	G/T	Forward	tctctctctcaactgactaaactaggtgccacgtcgtgaaagtctgacaaAAGAGCTATAAGAGCTGAGATC
X143	73	A/C	Reverse	ctctctctcaactgactaaactaggtgccacgtcgtgaaagtctgacATAAGATTAATAGTTTCAGGCACTG
X029	77	A/G	Forward	ctctctctctctctctcaactgactaaactaggtgccacgtcgtgaaagtctgacaaATTGGTTCTTCAGTCCCTC
X085	81	A/G	Reverse	tctctctctctctctctctcaactgactaaactaggtgccacgtcgtgaaagtctgacaaAGGAAAATGAGGATACCAAG
X009	85	A/G	Reverse	tctctctctctctctctctcaactgactaaactaggtgccacgtcgtgaaagtctgacaTGTTTCCAGAAGACCTAGTATTTTG

Lower case letters: Neutral sequence tail.

Upper case letters: Target-specific sequence.

### Statistics

Allele and haplotype frequencies were estimated by direct gene counting. Intrapopulational genetic diversity parameters were computed with ARLEQUIN 3.1 [41]. Gene diversity (d), equivalent to the expected heterozygosity for diploid data, was calculated as: $\text{d}=\frac{n}{n-1}\left(1-\sum_{1}^{k}p_{i}^{2}\right)$, where *n *is the number of gene copies, *k *the number of haplotypes and *p*_*i *_the sample frequency of the i^th ^allele at the locus [42]. The significance of an association between pairs of loci was tested using an exact test of significance [41], with 100,000 steps in the Markov chain and 10,000 dememorization steps. The procedure is analogous to Fisher's exact test. The resulting p values were corrected with the step-down Holm-Sidak procedure: *p*_*s *_= 1 - (1 - *pi*)^(*n*-*i*+1)^, where p_s _is the corrected p value, p_i _is i^th ^p value of all the values sorted increasingly, and n is the number of pairwise comparisons [43]. The null hypothesis of linkage equilibrium from multilocus data was tested using a parametric method implemented in the LIAN 3.5 software [44]. In order to evaluate the amount of population genetic differentiation, the 13 populations selected for this study and the four populations from the HapMap collection were analyzed. For some of the analyses, the 17 populations were re-organized in two different groups: the "Mediterranean" group, which included 11 populations (Catanzaro, Cosenza, Ibiza, Iraqi, Majorcan, Moroccan, Calabrian, Sicilian, Tunisian, Turkish and Valencia), and the "worldwide" group with nine populations from three continents: Europe (Danes, CEU and Calabrian), Africa (Moroccans, YRI and Somalis) and Asia (Iraqis, CHB and JPT).

An AMOVA analysis with two hierarchical levels was performed using ARLEQUIN 3.1. In this case, F_st _was calculated as $F_{st}=\frac{\sigma_{a}^{2}}{\sigma_{T}^{2}}$, where σ_a_^2 ^is the covariance component due to differences among the populations, and σ_T_^2 ^is the total molecular variance calculated as the sum of the covariance components among haplotypes within a population, among haplotypes in different populations and the component originated by differences among populations. We also performed a three hierarchical level AMOVA using ARLEQUIN 3.1 when several groups of population were analyzed. F_st _was tested by permuting haplotypes among populations among groups; F_sc _was tested among populations within groups; and F_ct _was tested by permuting populations among groups. Pairwise F_st _values [45] were obtained for all population pairs. A principal coordinate plot was drawn from the distance matrix obtained. Negative *eigenvalues *were corrected using Lingoes method [46] by means of DistPCoA program [47].

Additionally, genetic structure at individual level was investigated using STRUCTURE v. 2.2 [19]. We performed a cluster analysis using the admixture model. A burn-in time of 200,000 steps, followed by another 200,000 steps of the Markov Chain for data collection was used. We carried out five independent replicates for each value of K (predefined number of inferred populations).

A demographic analysis was performed using Rousset's two-dimensional isolation-by-distance model [21] with the ISOLDE program implemented in GENEPOP software version 3.4 [48]. The significance of the Spearman rank correlation coefficient was calculated with 10^6 ^Mantel's permutations.

Finally, we carried out a comparison between the results obtained from the 25 X-chromosome SNPs with results obtained on mainly the same populations by using non-recombinant markers (35 SNPs on the Y-chromosome and mtDNA HVRI sequences). We used the results obtained from the analysis of 35 biallelic Y-chromosome SNPs carried out on the same set of samples used for the present work (unpublished data and [49]). We used mtDNA data on HVRI sequences obtained from the literature: Italy [50], Sicily [51], Valencia [15], Ibiza [15], Majorca [15], Tunisia [2], Morocco [52], Turkey [53], Denmark [29], Germany [54], Iraq [29] and Somalia [55].

The sequences between the nucleotide positions 16,024 and 16,366 were analyzed [56]. Due to possible sequencing artefacts, the poly C tract (nucleotide positions from 16,184 to 16,191) was not taken into account for the analysis.

The levels of correlations between the different distance matrices obtained from the three groups of markers (X-chromosome, Y-chromosome and mtDNA) were calculated by means of the Mantel test (ARLEQUIN 3.1 software). Because the correlation between genetic distance matrices can be blurred by the geographic distance between populations [57], we calculated the partial correlations at constant geographical distances (r(set of markers 1, set of markers 2), geographic distance)). The sex-biased migration was studied by comparing the results obtained on the three data sets analyzed under Rousset's isolation-by-distance model [21] as suggested [24].

## Authors' contributions

CT was primarily responsible for the design of the study, carried out the SNP typing, performed the statistical analysis and elaborated the manuscript. JJS participated in designing the study and the manuscript. AB participated in designing the study and the manuscript, and sent samples from South Italy and Sicily. CBC participated in designing the study and the manuscript, and sent samples from Tunisia. AH participated in designing the study and the manuscript, and sent samples from Morocco. MBD participated in designing the study and the manuscript, and sent samples from Tunisia. MR participated in designing the study and the manuscript, and sent samples from Spain. NM participated in designing the study, analyzing the results and preparing the manuscript. All authors read and approved the final version of the manuscript.
